# Supplementary material for: Functioning of the EROS-R Scale in a Clinical Sample of Psychiatric Patients: New Psychometric Evidence from the Classical Test Theory and the Item Response Theory
Source: Int J Environ Res Public Health. 2022 Aug 15;19(16):10062. doi: 10.3390/ijerph191610062 (PMC9407833; doi:10.3390/ijerph191610062)
Supplement: Supplementary file 1 [file ijerph-19-10062-s001.zip › ijerph-1805103-supplementary.pdf]

Environmental Reward Observation Scale (EROS-B)

Instrucciones:

Valore en qué grado son aplicables a usted las siguientes diez frases. Recuerde no hay respuestas correctas ni incorrectas, lo importante es que responda con la máxima sinceridad posible. No emplee mucho tiempo en cada pregunta.

Para responder utilice la siguiente clave:

TD	ED	DA	TA
Totalmente en desacuerdo	En desacuerdo	De acuerdo	Totalmente de acuerdo

**N°**	**Ítems**	**TD**	**ED**	**DA**	**TA**
1	Muchas actividades de mi vida son agradables				
2	Últimamente, me he dado cuenta que las experiencias que vivo, me hacen feliz				
3	En general, estoy muy satisfecho con la forma en que utilizo mi tiempo				
4	Me resulta fácil encontrar motivos para disfrutar de la vida				
5	Mi vida es tan gratificante como la de otras personas				
6	Las actividades que antes hacía me siguen resultando gratificantes				
7	Encuentro actividades que me divierten				
8	Estoy satisfecho con mis logros				
9	Mi vida es interesante				
10	Las actividades que realizo normalmente salen bien				
